# Aldehyde dehydrogenases inhibition eradicates leukemia stem cells while sparing normal progenitors

**DOI:** 10.1038/bcj.2016.78

**Published:** 2016-09-09

**Authors:** G Venton, M Pérez-Alea, C Baier, G Fournet, G Quash, Y Labiad, G Martin, F Sanderson, P Poullin, P Suchon, L Farnault, C Nguyen, C Brunet, I Ceylan, R T Costello

**Affiliations:** 1 Aix-Marseille Université, INSERM, UMR1090, TAGC Campus, Marseille, France; 2 Department of Hematology and Cellular therapy, AP-HM, Conception Hospital, Marseille, France; 3 Advanced BioDesign, Lyon, France; 4 Lab Animal Models and Cancer Laboratory Anatomy Pathology Program, Institut de Recerca Valld' Hebron, Barcelona, Spain; 5 Univ Lyon, Université Claude Bernard Lyon 1, ICBMS UMR5246, Villeurbanne, France; 6 Department of Apheresis and Autotransfusion, APHM, Conception Hospital, Marseille, France; 7 Hematology laboratory, APHM, Hôpital de la Timone, Marseille, France; 8 UMR 1062 NORT, INSERM, Marseille, France; 9 Hematology laboratory, APHM, Hôpital de la Conception, Marseille, France

## Abstract

The vast majority of patients with acute myeloid leukemia (AML) achieve complete remission (CR) after standard induction chemotherapy. However, the majority subsequently relapse and die of the disease. A leukemia stem cell (LSC) paradigm has been invoked to explain this failure of CR to reliably translate into cure. Indeed, LSCs are highly enriched in CD34+CD38− leukemic cells that exhibit positive aldehyde dehydrogenase activity (ALDH+) on flow cytometry, these LSCs are resistant to currently existing treatments in AML such as cytarabine and anthracycline that, at the cost of great toxicity on normal cells, are highly active against the leukemic bulk, but spare the LSCs responsible for relapse. To try to combat the LSC population selectively, a well-characterized ALDH inhibitor by the trivial name of dimethyl ampal thiolester (DIMATE) was assessed on sorted CD34+CD38− subpopulations from AML patients and healthy patients. ALDH activity and cell viability were monitored by flow cytometry. From enzyme kinetic studies DIMATE is an active enzyme-dependent, competitive, irreversible inhibitor of ALDH1. On cells in culture, DIMATE is a powerful inhibitor of ALDHs 1 and 3, has a major cytotoxic activity on human AML cell lines. Moreover, DIMATE is highly active against leukemic populations enriched in LSCs, but, unlike conventional chemotherapy, DIMATE is not toxic for healthy hematopoietic stem cells which retained, after treatment, their self-renewing and multi-lineage differentiation capacity in immunodeficient mice, xenografted with human leukemic cells. DIMATE eradicates specifically human AML cells and spares healthy mouse hematologic cells.

## Introduction

Acute myeloid leukemia (AML) is the most common acute leukemia in adults, with a median age of 69 years.^1^ The vast majority of patients with AML achieve complete remission after standard induction chemotherapy. However, the majority subsequently relapse and die of the disease.^2, 3, 4^ A leukemia stem cell (LSC) paradigm may explain this failure of complete remission to reliably translate into cure. LSCs, like normal hematopoietic stem cells (HSCs), have self-renewal capacity and give rise to partially differentiated progeny that composes the bulk of the leukemia, but possesses only limited proliferative potential.^5^ The currently existing treatments in AML, such as cytarabine (ara-C) and anthracycline (for example, daunorubicin), at the cost of a great toxicity, are highly active against the leukemic bulk, but spare the LSCs responsible for relapse.^6, 7^ Therefore, AML remains a clinical challenge and new therapies are urgently needed.^8, 9, 10^

Only a rare population of AML cells enriched for LSCs, characterized by a CD34+CD38− phenotype is capable of generating leukemia in immunodeficient mice.^11^ More recently, evidence has been presented for a clinically relevant population of leukemic cells CD34+CD38− in AML. This leukemic subpopulation, with a positive aldehyde dehydrogenase activity (ALDH+) in flow cytometry has been shown to be highly enriched in LSCs.^12, 13, 14^ Interest in ALDH is due to its activity as a marker for identification of stem cell in different tissues.^15, 16^ The different isoforms of ALDHs (ALDH1, 2 and 3) control the levels of three endogenous apoptogenic aldehydes: methional, malondialdehyde (MDA) and 4-hydroxynonenal (HNE). Cancer cells protect themselves from the apoptogenic effect of these aldehydes by the ALDHs that oxidize them to their non-apoptogenic carboxylic acids.^17^

Among the family of acetylenic ALDH inhibitors, we identified the dimethyl ampal thiolester (DIMATE), an α,β, acetylenic N-substituted aminothiol ester, as an interesting candidate for cancer treatment. DIMATE is an active enzyme-dependent, competitive, irreversible inhibitor of ALDHs 1 and 3.^18, 19^ It induces apoptosis in the chemoresistant mouse lymphoid cells BAF3bcl2 that are also resistant to disulfiram, a well-characterized inhibitor of ALDH2.^20^ Moreover, although DIMATE was apoptogenic on cultures of human prostate cancer cells DU145, it was reversibly cytostatic on normal human prostate epithelial cells.^19^

On the basis of these preliminary data on ALDH activity in LSC and differential effects between normal and cancer cells, we hypothesized that DIMATE could be a candidate for targeted therapy on LSC while sparing normal hematopoietic progenitors, thus providing an efficient and safe approach for chemotherapy of acute leukemia aiming at the eradication of minimal residual disease.

## Materials and methods

### Patient samples

Peripheral blood samples from 10 patients with AML (Table 1) were collected before leukemia chemotherapy and after informed consent, and were part of the diagnostic procedures. The study was approved by the institutional review board from the Mediterranean V (Ref. 15.013) and Agence National de la Sécurité du Médicament (Ref. 150054B–11). Control non-leukemic HSCs were collected by apheresis from patients (*n*=55) requiring autologous stem cell transplantation for non-myeloid malignancies.

### Cell sorting

Peripheral blood mononuclear cells from control donors and leukemic patients were isolated by Ficoll-Hystopaque density gradient centrifugation (Sigma-Aldrich, Saint-Quentin, France). CD34+ cell sorting was first performed with CD34 MicroBead Kit UltraPure (MACS; Miltenyi Biotec, Paris, France). Then leukemic and healthy CD34+CD38− or CD34+CD38+ subpopulations were obtained by flow cytometry cell sorting using double staining with anti-CD34 (APC MACS; Miltenyi Biotec) and anti-CD38 (FITC, MACS; Miltenyi Biotec) mAbs, with an exclusion of at least 20 channels between the CD38+ and CD38− subpopulations (data not shown). The purity of the preparation (⩾99% of CD34+CD38− leukemic or healthy cells) was assessed by flow cytometry reanalysis of sorted cells. CD34+CD38− enriched cells were plated during 48 h at 6.10^4^ cells/ml in CellGro GMP SCGM medium supplemented with rh SCF (100 ng/ml), rh TPO (20 ng/ml) and rh Flt3 (50 ng/ml) (all from CellGenix GmbH, Freiburg, Germany).

### Cell culture

We used the following leukemic cell lines: HL-60 (derived from a 36-year-old female with (AML M2), THP-1 (derived from a 1-year-old male with AML M5), Kasumi-1 (derived from a 7-year-old male with AML M2), Kasumi-3 (derived from a 57-year-old male with AML M0), MOLM-14 (derived from a 20-year-old male with AML M5) and KG-1(derived from a 59-year-old male with AML M6). All AML cells lines were obtained from the American Type Culture Collection (ATCC), the European Collection of Cell Cultures (ECACC) and were cultured in RPMI-1640 Glutamax supplemented with 10–20% fetal bovine serum (GE Healthcare fetal bovine serum, Europe, GmbH, Pasching, Austria) at 37 °C in a humidified atmosphere of 5% CO_2_–95% air.

### Cell viability

Cells were seeded into 96-well cell culture plates at a concentration of 50 000 cells/well. Cell viability was monitored by AlamarBlue Cell viability protocol (Thermo Fisher Scientific, Illkirch, France) on a TriStar LB 941 Multimode Microplate Reader (Berthold Technologies, Thoiry, France). The sensitivity toward different drugs DIMATE (Advanced BioDesign, Lyon, France), cytarabine, daunorubicine and azacytidin (Sigma-Aldrich) was determined using different concentrations of the drugs. After 48 h, the growth inhibitory effect of the drug was analyzed using Resasurin (Sigma-Aldrich) according to manufacturer's instruction. The drug response was quantified by the half maximal inhibitory concentration (IC50) for each particular cell line, and determined by non-linear regression analysis of log-dose/response curves.

ALDH activity (ALDEFLUOR kit, StemCell Technologies, Grenoble, France) and cell viability (with 7-AAD, Immunotech, Marseille, France), of the healthy and leukemic CD34+CD38−, were concomitantly performed by flow cytometry, to monitor specifically the CD34+CD38-ALDH+ and CD34+CD38-ALDH− subpopulations viability.

### ALDEFLUOR assay

Enzyme activity of ALDH was detected with ALDEFLUOR kit (StemCell Technologies). Control and leukemic CD34+CD38− cells and leukemic cell lines (1.10^6^ cells/ml) were incubated under control condition or treated with 1,3,5 10 μmol/l of DIMATE overnight. Then, cells were washed in phosphate-buffered saline (PBS) 1 × and were suspended in ALDEFLUOR assay buffer. For each condition (250 000 cells/500 μl), diethyl aminobenzaldehyde (DEAB), a broad inhibitor of ALDH, was used as control of the background fluorescence. ALDEFLUOR DEAB reagent (‘control' condition) or ALDEFLUOR activated reagent (‘test' condition) were added to cell suspension and were incubated 30 min at 37 °C.

### Quantitative determination of HNE protein and MDA/protein adducts by ELISA

The formation of 4-HNE protein and MDA protein adducts was quantified with the Oxiselect HNE Adduct Elisa kit (Cell Biolabs, San Diego, CA, USA) and the OxiSelect MDA Adduct ELISA Kit (Cell Biolabs), respectively. Briefly, cell lysates were prepared by sonication in reducing SDS Sample Buffer. Homogenates were diluted to 10 μg protein/ml and adsorbed in 96-well protein binding plates by incubation at 37 °C for at least 2 h. Wells were washed twice with PBS and incubated for an additional 2 h at room temperature on an orbital shaker. Following three washes in PBS, 100 μl of anti-HNE antibody or anti-MDA were added to the wells and incubated for 1 h at room temperature. Subsequently, goat anti-rabbit secondary antibody-HRP conjugate (diluted 1/1000 with the assay diluent) was added and incubation continued for 1 h. Wells were washed five times in PBS and HRP substrate was added. Reaction was stopped with an acidic solution, and absorbance read on a microplate reader at 450 nm. The amount of HNE protein adducts was determined by comparison with a standard curve prepared from HNE-BSA and MDA-BSA standards supplied by the manufacturer.

### Caspase activity assay

The caspase activity assay on DIMATE-treated HL-60 cells was evaluated using the Caspase-Glo 3/7 Assay (Promega, Madison, WI, USA) following the manufacturer's instructions. HL-60 cells were seeded at a concentration of 5 × 10E4 cells/well in 100 μl of complemented medium into 96-well flat-bottom white microplates. Cells were grown in presence of DIMATE 5 μmol/l or vehicle in triplicate. After 1, 3, 6 and 24 h of incubation, 100 μl of Caspase-Glo Reagent was added and the mixture was incubated for 1 h at 37 °C. The amount of luminescent corresponding to the activity of caspase 3 and 7 was quantified using a luminometer.

### Colony-forming units

Human HSCs were cultured under control conditions or with 1, 3, 5, 7, 5, 10 and 15 μmol/l of DIMATE. To observe the self-renewing hematopoietic system, cells were washed with PBS 1 × and then counted in Cellgenix medium (CellGenix GmbH) supplemented with cytokines. About 1000 to 1500 cells were plated in dishes in StemMACS HSC-CGU Media (MACS; Miltenyi Biotec) in a 37 °C humidified atmosphere containing 5% CO_2_. The clonogenic assays were performed in triplicate. Fifteen days later, colonies coming from differentiation of progenitors cells were counted and evaluated accordingly to manufacturer's instructions. Colony-forming unit-granulocyte macrophage, erythroid and burst-forming unit-erythroid and megakaryocyte progenitors were identified.

### *In vivo* experimentation

To determine the antileukemic activity of DIMATE in a clinically relevant setting, we established patient-derived xenografts, or PDX models, in which 3 × 10^6^ immunopurified CD34+ leukemic peripheral blood mononuclear cells from AML patient were transplanted intravenously into NOD/SCID/IL2Rγ^null^ immunodeficient mouse strain (NOG) for expansion (*n*=25). For human AML xenograft, a patient with refractory AML after induction (complex karyotype+del(5q), bad prognosis, see UPN2, Table 1) was selected. After injection of primary AML cells, NOG mice were monitored for leukemia development by flow cytometric analysis of peripheral blood for human CD45-positive (hCD45+) cells. Four weeks were required for the development of the models monitored by the h-rate corresponding to the amount of hCD45+cells/mouse CD45-positive cells (mCD45+). Mice were next randomized and treatment with DIMATE (14, 28 mg/kg) or drug vehicle started for 4 weeks. Weekly monitoring of hCD45+ and mCD45+ circulating cells was performed during all treatment period. After treatment, mice were killed and bone marrow and spleen were harvested. hCD45+ and mCD45+ sorting and monitoring, in spleen and bone marrow, and spleen weighing was performed. All *in vivo* animal studies had been reviewed and approved by the local ethics committee (01_TransCurebioServices-AB-01).

### Statistical analysis

Values are expressed as mean±s.e.m. or frequencies and proportions. Differences between the groups were determined by unpaired *t*-test, *χ*^2^-, Fisher's exact test or analysis of variance, where appropriate. *P*<0.05 was considered statistically significant. Analysis was performed using GraphPad prism version 5.0 (GraphPad software, San Diego, CA, USA).

## Results

### Inhibition of ALDH by DIMATE is cytotoxic on human AML cell lines by promoting apoptogenic aldehyde accumulation inducing apoptosis

**Figure 1:**
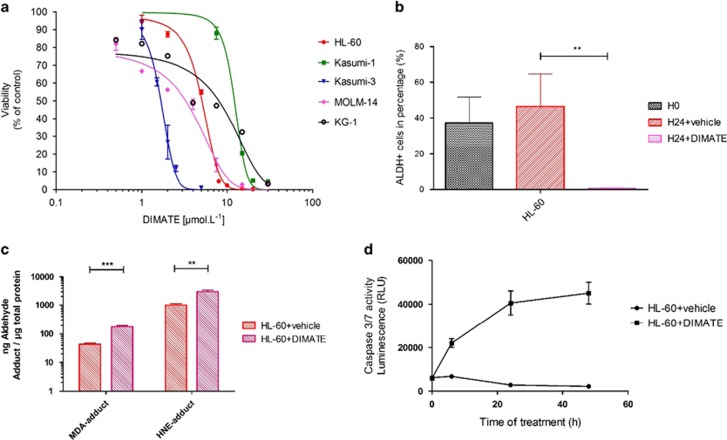
Inhibition of ALDH by DIMATE is cytotoxic on human AML cell lines by promoting apoptogenic aldehyde accumulation inducing apoptosis. (**a**) Viability assay of AML cells to increasing concentration of DIMATE. All experiments were performed in triplicates (*n*=3). Values for all cell lines are showed in Supplementary Table 2. (**b**) Monitoring of aldehyde dehydrogenase activity in flow cytometry in HL-60. Graphic representations of average percentages of ALDH+ cells at H0, and after 24 h of cell culture, without and with DIMATE 5 μmol/l. (*n*=3). (**c**) Quantification of MDA and HNE adduct in HL-60 cells treated with DIMATE 5 μmol/l during 24 h. Adduct formation is higher in cells treated than with the vehicle. (**d**) Monitoring of caspase 3/7 activity of HL-60 cells treated with DIMATE 5 μmol/l. After 6 h of treatment, activation of caspase activity is observed.

DIMATE's IC50 was determined in AML cell lines showing a cytoxic effect of DIMATE with IC50 ranged between 1 and 15 μm. On HL-60, Kasumi-1, Kasumi-3, MOLM-14 and KG-1 cell lines, the IC50 of DIMATE was of 5.094 μmol/l (s.e.m.±0.1007); 12.2 μmol/l (s.e.m.±0.4711); 1.67 μmol/l (s.e.m.±0.04758); 3.287 μmol/l (s.e.m.±0.2039) and 8.028 μmol/l (s.e.m.±1.386), respectively (Figure 1a and Supplementary Table 1).

Cytotoxic effect of DIMATE is due to the inhibition of ALDH activity that leads to accumulation of apoptogenic aldehydes such as MDA and HNE. Treatment of AML cell, HL-60 with DIMATE for 24 h shows an inhibition of ALDH activity (Figure 1b) and accumulation of HNE and MDA adducts bound to proteins (Figure 1c). This accumulation caused apoptosis in a time-dependant manner by activating caspases 3 and 7 (Figure 1d).

### DIMATE is selectively cytotoxic in leukemic population enriched in LSCs but unlike conventional chemotherapy, DIMATE is not toxic for healthy hematopoietic stem cells

**Figure 2:**
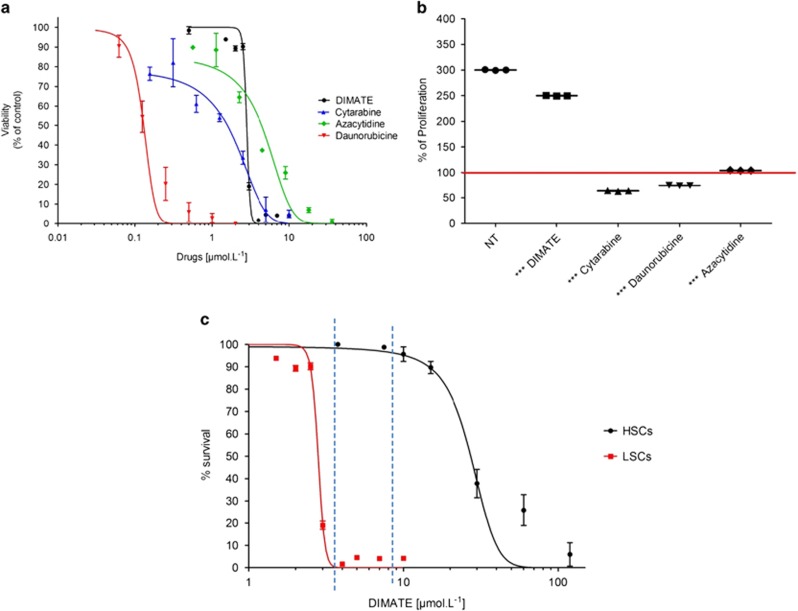
Cytotoxicity profile of DIMATE, daunorubicine, cytarabine and azacytidine on CD34+CD38−ALDH+ leukemic cells and CD34+CD38− healthy hematopoietic stem cell. (**a**) Cytotoxicity profiles of DIMATE, daunorubicine, cytarabine and azacytidine after 48 h of treatment for the CD34+CD38−ALDH+ leukemic cells population enriched in LSCs (*n*=10). (**b**) Healthy HSCs proliferation, in percentage, after 48 h of treatment with DIMATE, daunorubicine, cytarabine and azacytidine. Drugs were used at a concentration equal to the IC50 values determined for the different drugs in LSCs (*n*=51). (**c**) LSCs and healthy HSCs survival according to the different concentration of DIMATE (*n*=10). Doted lines (5–9 μmol/l) determine a therapeutic window within DIMATE eradicated all LSCs (100% of lethality) and showed low toxicity (under 3% of lethality) on normal HSCs.

The cytotoxicity of DIMATE was assessed on CD34+ CD38− ALDH+ leukemic peripheral blood mononuclear cells. For this population, enriched for LSCs, IC50 values of DIMATE was 2.8 μmol/l (s.e.m.±0.03). For this same leukemic population, IC50 values of daunorubicin, ara-C and azacytidine were respectively 0.132 μmol/l (s.e.m.±0.009), 1.5 μmol/l (s.e.m.±0.17) and 4.41 μmol/l (s.e.m.±0.4) (Figure 2a and Supplementary Table 2).

Healthy HSCs were treated for 48 h with the IC50 values of DIMATE, daunorubicin, ara-C and azacytidine determined on the LSCs. After 48 h of HSCs culture, mean proliferation between H0 and H48 was 300% (s.e.m.±4.398) in the untreated group and 250% (s.e.m.±3.665) in the DIMATE group (*P*<0.01). In the daunorubicin and ara-C groups there was no HSCs proliferation but a cellular death respectively of 20% (s.e.m.±4.691) (*P*<0.01) and 40% (s.e.m.±3.417) (*P*<0.01). After 48 h of treatment, azacytidine had almost a cytostatic effect on HSCs (*P*<0.001) (Figure 2b). Figure 2c shows cell survival for the LSCs and HSCs according to the different concentrations of DIMATE. The IC50 concentration was 2.79 μmol/l (s.e.m.±0.04) for the LSCs and 24.39 μmol/l (s.e.m.±1.58) for HSCs (*P*<0.01). A therapeutic zone could be established. Indeed, between 5 and 9 μmol/l, DIMATE eradicated all LSCs (100% of lethality) and showed low toxicity (under 3% of lethality) on healthy HSCs.

Moreover, after 48 h of cell culture with DIMATE until 10 μmol/l, normal HSCs retained their self-renewing and multi-lineage differentiation capacity as shown by the enumeration and evaluation of stem and progenitor cells as colony-forming units in the Supplementary Figure 1. Above 10 μmol/l, HSCs self-renewing and multi-lineage differentiation capacity significantly and drastically decreased (*P*<0.01). No colony-forming units were observed above 15 μmol/l (data not shown).

### In immunodeficient mice, xenografted with human leukemic cells, DIMATE eradicates specifically human AML cells and spares healthy mouse hematologic cells

**Figure 3:**
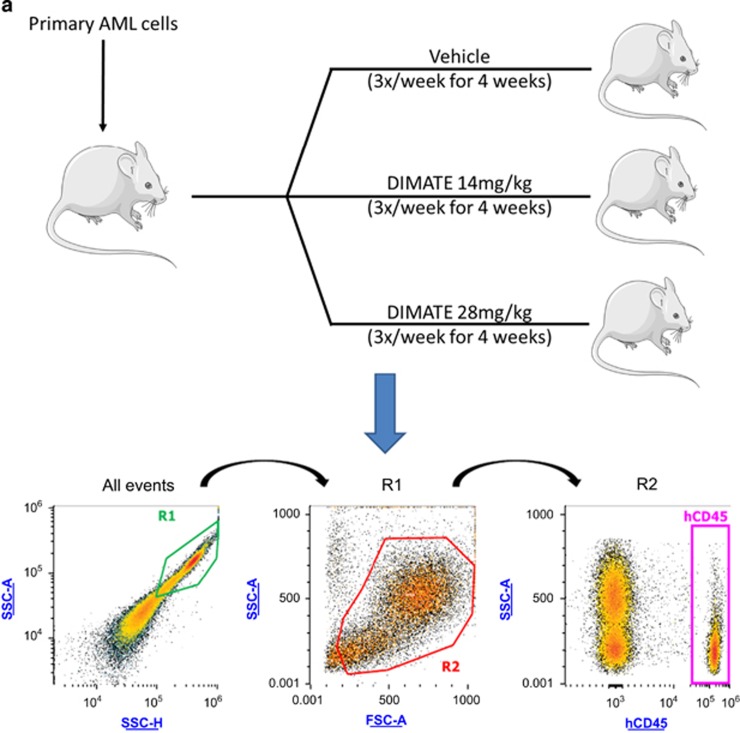

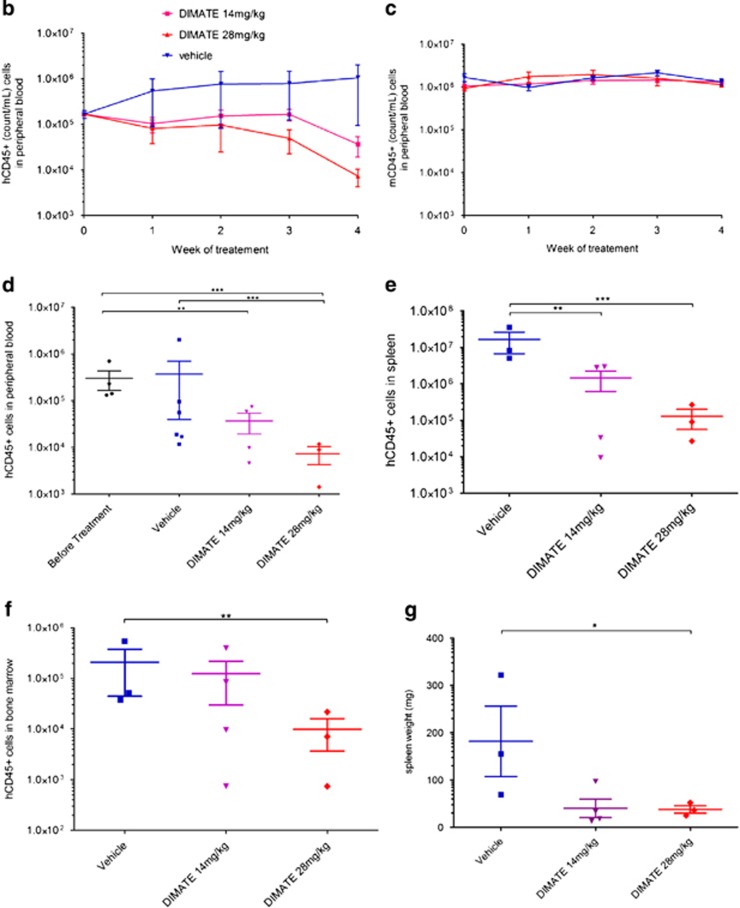
DIMATE shows potent activity against primary AML cells transplanted in NOG mice. (**a**) Experimental scheme and gaiting strategy in flow cytometry for hCD45+ and mCD45+ cells monitoring in blood, spleen and bone marrow for study of the antileukemic activity of DIMATE in immunodeficient mice engrafted with primary human AML cells. Mice were randomized and treatment with DIMATE (14, 28 mg/kg) and vehicle started for 4 weeks. Weekly monitoring of hCD45+ and mCD45+ circulating cells were performed during the treatment. After treatment, mice were killed and bone marrow and spleen were harvested. CD45+ have been sorted and monitored in spleen and bone marrow. Spleen weighing was performed. (**b**) Human CD45+ cells (hCD45+) count in peripheral blood during the treatment with vehicle or DIMATE. (**c**) Mouse CD45+ cells (mCD45+) count in peripheral blood during the treatment with vehicle or DIMATE. (**d**) Absolute human AML cells in the peripheral blood of NOG mice engrafted with AML cells before and after treatment with vehicle and different concentrations of DIMATE after mice euthanasia. (**e**) Counts of human CD45 cells in spleen in mice engrafted with AML cells after treatment with vehicle and different concentrations of DIMATE. (**f**) Counts of human CD45 cells in bone marrow in mice engrafted with AML cells after treatment with vehicle and different concentrations of DIMATE. (**g**) Spleen weight of mice transplanted with human AML cells treated with vehicle or DIMATE. In panels (**d**–**f**) each symbol denotes a single animal (*n*=3–6 per group). ** Mean *P*<0.001 and * mean *P*<0.05.

NOG immunodeficient mice were engrafted with primary human AML cells. Four weeks post engraftment, hCD45+ cells represented 17±14% of circulating cells (data not shown). Mice were next randomized and treatment with DIMATE (14, 28 mg/kg) and vehicle started for 4 weeks (Figure 3a). In untreated control mice (vehicle), after 4 weeks, hCD45+ AML circulating cells increased from 3 × 10^5^ (s.e.m.±1.3 × 10^5^) to 3.7x10^5^ (s.e.m.±3.3 × 10^5^) (not significant). In contrast, in 14 and 28 mg/kg DIMATE-treated mice, there was a drastic and significant decrease in human AML (hCD45+) cells compared with those in the untreated mice from 3 × 10^5^ (s.e.m.±1.3 × 10^5^) to 3.7 × 10^4^ (s.e.m.±1.7 × 10^4^) in the DIMATE 14 mg/kg group (*P*<0.01) and from 3 × 10^5^ (s.e.m.±1.3 × 10^5^) to 7.3 × 10^3^ (s.e.m.±3 × 10^3^) in the DIMATE 28 mg/kg group (*P*<0.01) (Figures 3b and d). Furthermore, during all of the treatment period, no toxicity or decrease in healthy circulating mouse cells (mCD45+) was observed in all the groups, including the DIMATE 14 and 28 mg/kg groups (Figure 3c). After 4 weeks of treatment, mice were killed and bone marrow and spleen were harvested. CD45+ sorting and monitoring, in spleen and bone marrow, but also spleen weighing was performed. Compared with untreated control mice, in the DIMATE 14 and 28 mg/kg treatment groups, spleen infiltration by human AML cells drastically decreased from 1.7 × 10^7^ (s.e.m.±9.9 × 10^6^) to 1.4 × 10^6^ (s.e.m.±8.2 × 10^5^) in the DIMATE 14 mg/kg group (*P*<0.01) and to 1.3 × 10^5^ (s.e.m.±7.2 × 10^4^) in the DIMATE 28 mg/kg group (*P*<0.01) (Figure 3e). At the same time, each mouse spleen was weighed. Compared with untreated mice, spleen weight decreased from182 (s.e.m.±74.2 g) to 40.25 g (s.e.m.±19.4 g) in the DIMATE 14 mg/kg group (not significant)) and to 37.7 g (s.e.m.±7.9 g) in the DIMATE 28 mg/kg group (*P*<0.05) (Figure 3g). Regarding bone marrow, compared with untreated control mice, infiltration by AML cells decreased from 2.1 × 10^5^ (s.e.m.±1.7 × 10^5^) to 1.2x10^5^ (s.e.m.±9.3 × 10^4^) in the DIMATE 14 mg/kg group (not significant) and to 9.8 × 10^3^ (s.e.m.±6.2 × 10^3^) in the DIMATE 28 mg/kg group (*P*<0.05) (Figure 3f).

## Discussion

Resistance to cytarabine and anthracycline-based chemotherapy is a major cause of treatment failure in AML.^10^ More precisely, CD34+CD38− leukemic cell population, enriched in LSCs, are highly resistant to these and other conventional chemotherapies.^21^ Therefore new therapies are urgently needed for this deadly disease. Furthermore, conventional chemotherapy have similar cytotoxic effects on normal or leukemic HSCs.^21^ In sharp contrast, we demonstrated that DIMATE, through ALDH inhibition, targeted and eradicated *in vitro* and *in vivo* several human myeloid leukemia cell lines and human leukemic cells population highly enriched in LSCs, but spared healthy HSCs. Most interestingly, we have determined a therapeutic zone, between 5 and 9 μmol/l, where DIMATE eradicated all LSCs (100% of lethality) and showed low toxicity (under 3% of lethality) on healthy HSCs. Moreover, with such treatment concentrations, healthy HSCs retained their self-renewing and multi-lineage differentiation capacity.

ALDH detoxifying enzymes has been extensively associated with a number of malignancies and drug-resistant phenotypes. In the particular case of AML, the ALDH gene family is found upregulated or amplificated in 42% of cases, impacting also life expectancy, negatively (Supplementary Figures 2A and B).^22, 23^ These data suggest that the altered ALDH activity in AML confers advantages for cell proliferation and survival and/or for the progression of the disease. DIMATE is described as an irreversible inhibitor of recombinant ALDH1 and ALDH3. Therefore, we evaluated the capacity of DIMATE to inhibit the ALDH activity in leukemic cells.^18, 19^

Selective cytotoxicity of DIMATE on cancer cells of human and murine origin has already been demonstrated *in vitro* on human epithelial cancer cells. Indeed, DIMATE induces an irreversible apoptosis in human prostate epithelial cancer cells DU145, but it is a reversible cytostatic agent on human prostate epithelial normal cells.^18, 19^ In the specific AML framework, safety of ALDH inhibitor on healthy HSCs has already been demonstrated, and even more remarkable is the inhibition of ALDH and retinoid signaling induces expansion of human HSCs.^24^

These hypotheses are supported by our experiments in mice. In NOG mice, xenografted with human AML, enriched in LSCs, DIMATE eradicated *in vivo*, specifically, human AML cells (hCD45+) in blood, spleen and bone marrow. In contrast, DIMATE spared healthy circulating mouse cells (mCD45+). Moreover, with a humanization rate of 17%, our mouse model could be optimized, to try to obtain more significant results, in spleen and bone marrow, in particular, in the DIMATE 14 mg/kg treatment group.

With very unusual anti-cancer properties, DIMATE seems to be a very promising molecule for the treatment of AML and cancer more generally. Results from our work open new therapeutic perspectives in AML and provide a conceptual support for initiation of a phase I–II clinical trials.

We conclude that DIMATE is a very promising drug that opens new therapeutic perspectives in myeloid malignancies with putative interest in lymphoid malignancies as this drug is also able to inhibit the anti-apoptotic effect of bcl-2.^18^ A limitation of our work concerns the LSC definition. Indeed, in our work, to define LSCs we used the CD34+CD38−ALDH+ phenotype. However, several pathways, specific genes or microRNA, cytometric or transcriptomic signatures have also been proposed to distinguish LSCs from HSCs.^25, 26, 27, 28^ There is no consensus on an absolute definition for LSCs and it is more correct to speak of 'leukemic cells population enriched in LSCs'. Furthermore, according to some robust experimental works, LSCs could not be a stem cell disorder but rather a reacquisition of stem cell characteristics by classic leukemic cells.^29^ Finally, we should not underestimate the role of the bone marrow environment in the leukemogenesis. Indeed, a pathologic bone marrow ‘niche' could lead to permanent generation of LSCs.^30, 31, 32^ Nevertheless, LSCs remain an interesting target for other innovative therapeutic strategies.^33, 34, 35^ Analysis of DIMATE effect on normal and leukemic bone marrow ‘niche' is the next step in our study aiming at a better understanding of the mechanisms of action of this innovative drug.

## Figures and Tables

**Table 1:** AML patients and their clinical characteristics

*AML*	*Age*	*Sex*	*Type*	*Circulating blasts*	*De novo or secondary AML*	*Previous treatments*	*Cytogenetic*
UPN1	74	F	AML1	60%	Secondary to myelodysplastic syndrome	5 cycles of azacitidine	45,XX,+del (7q)
UPN2	79	F	AML1	81%	*De novo*	Induction+consolidation elderly patients (cytarabine–idarubicin)	Complex karyotype+del(5q)
UPN3	77	M	AML1	83%	*De novo*	Standard induction (daunorubicin–cytarabine)	Normal karyotype
UPN4	64	F	AML0	25%	Secondary to primary myelofibrosis	Double induction (daunorubicin–cytarabine followed by cytarabine–idarubicin)	Complex karytoype
UPN5	75	M	AML0	40%	Secondary to myelodysplastic syndrome	Untreated	Complex karyotype+del (5q)+del (7q)
UPN6	73	F	AML5	22%	*De novo*	Induction+consolidation elderly patients (cytarabine–idarubicin)	t (6;11) MLL-AF6
UPN7	71	M	AML5	34%	Secondary to myelodysplastic syndrome	Induction+consolidation elderly patients (cytarabine–idarubicin)	Del (5q), del(7q), del(17p)
UPN8	91	F	AML0	59%	*De novo*	Untreated	Unknown
UPN9	48	M	AML7	27%	Secondary to chronic myelomonocytic leukemia	2 cycles of azacitidine+2 cycles of decitabine	Normal karyotype
UPN10	78	M	AML5	80%	*De novo*	Untreated	Normal karyotype

Abbreviations: AML, acute myeloid leukemia; UPN, unique patient number.
