# Supplementary material for: Aldehyde dehydrogenases inhibition eradicates leukemia stem cells while sparing normal progenitors
Source: Blood Cancer J. 2016 Sep 9;6(9):e469–. doi: 10.1038/bcj.2016.78 (PMC5056970; doi:10.1038/bcj.2016.78)
Supplement: Supplementary Figure 1 [file bcj201678x1.doc]

**Supplementary Figure 1. Colony Forming Unit (CFU) Assay performed in CD34+ cells from 3 healthy donors.** Each data point represents the average of 3 samples with triplicate plating normalized to vehicle control. After 48 hours of treatment by DIMATE, healthy CD34+ were washed and clonogenic assays were performed. CFU count was realized 15 days after DIMATE treatment. Significant decrease was observed in the CFU-E, CFU-GEMM treated with 7.5µmol/L of DIMATE and in all CFU with 10 µmol/L of DIMATE in comparison with untreated CD34+ (p <0.05).

**
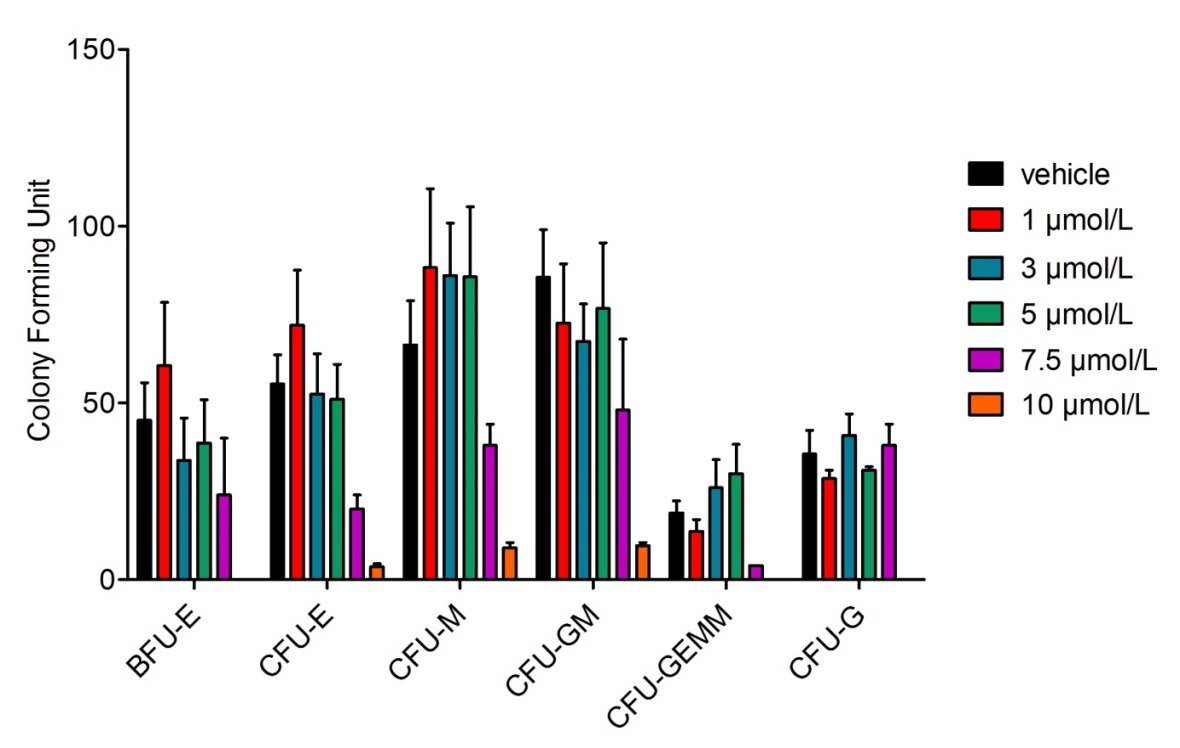
**

**eee**
