# Supplementary material for: Aldehyde dehydrogenases inhibition eradicates leukemia stem cells while sparing normal progenitors
Source: Blood Cancer J. 2016 Sep 9;6(9):e469–. doi: 10.1038/bcj.2016.78 (PMC5056970; doi:10.1038/bcj.2016.78)
Supplement: Supplementary Figure 2 [file bcj201678x2.doc]

**Supplementary Figure 2.** **(Data from cBioportal data base). Genetic alteration of ALDH genes family in 166 AML patients. A,** Genetic alteration of ALDH genes in 166 AML patients. **B,** Overall survival in month in AML patients with (in red) or without (in blue) genetic alteration in ALDH genes.

**A**

**
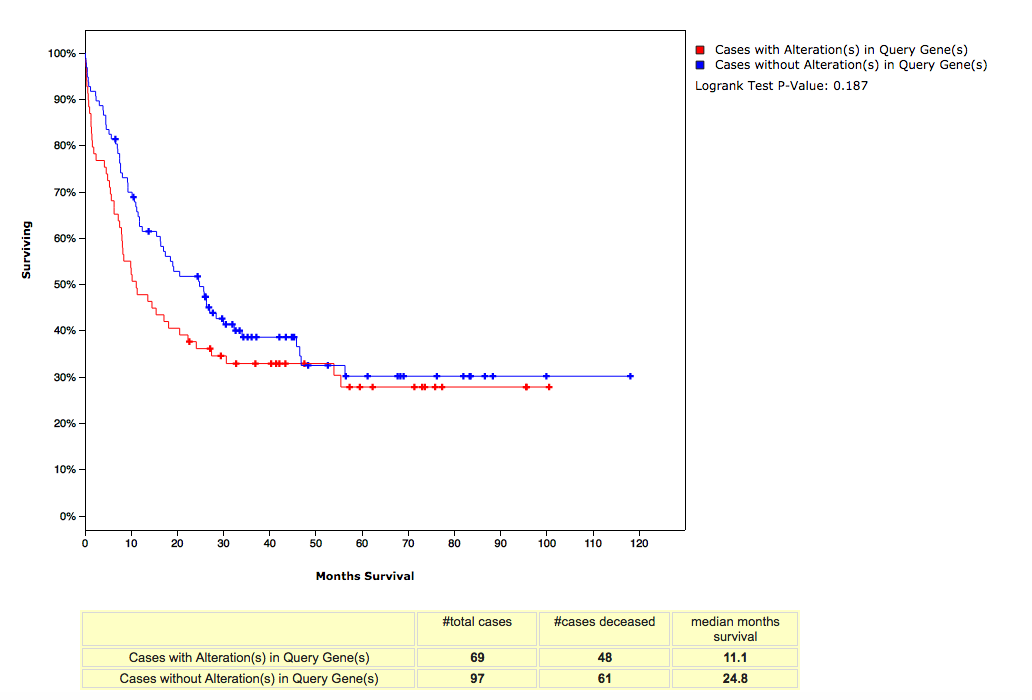

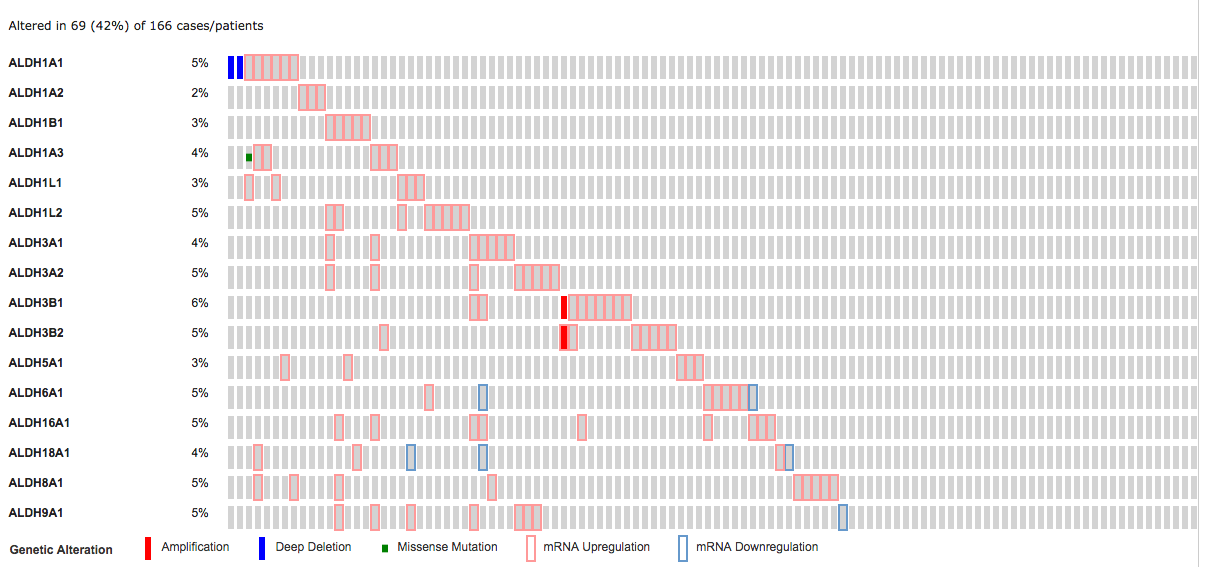
**

**B**
