# Supplementary material for: Aldehyde dehydrogenases inhibition eradicates leukemia stem cells while sparing normal progenitors
Source: Blood Cancer J. 2016 Sep 9;6(9):e469–. doi: 10.1038/bcj.2016.78 (PMC5056970; doi:10.1038/bcj.2016.78)
Supplement: Supplementary Table 1 [file bcj201678x3.doc]

**Supplementary Table 1.** IC50 values of DIMATE (with SEM), in the following leukemic cell populations: HL-60, Kasumi-1, Kasumi-3, MOLM-14 and KG-1. (n=6)

|  | **HL-60** | **Kasumi-1** | **Kasumi-3** | **MOLM-14** | **KG-1** |
| --- | --- | --- | --- | --- | --- |
| **IC50 (µmol.L^-1^)** | 5.094 | 12.2 | 1.67 | 3.287 | 8.028 |
| **SEM (±)** | 0.1007 | 0.4711 | 0.04758 | 0.2039 | 1.386 |
