# Supplementary material for: Aldehyde dehydrogenases inhibition eradicates leukemia stem cells while sparing normal progenitors
Source: Blood Cancer J. 2016 Sep 9;6(9):e469–. doi: 10.1038/bcj.2016.78 (PMC5056970; doi:10.1038/bcj.2016.78)
Supplement: Supplementary Table 2 [file bcj201678x4.doc]

**Supplementary Table 2.** IC50 values (with SEM) of DIMATE, daunorubicine, cytarabine and azacytidine for CD34+CD38-ALDH+ leukemic cells population enriched in LSCs.

|  | **DIMATE** | **Cytarabine** | **Daunorubicine** | **Azacytidine** |
| --- | --- | --- | --- | --- |
| **IC50 (µmol.L^-1^)** | 2.799 | 1.574 | 0.132 | 4.406 |
| **SEM (±)** | 0.032 | 0.166 | 0.009 | 0.413 |
